# Throat Disease

**Published:** 1869-01

**Authors:** 


### THROAT DISEASE.

Some years ago a poor young man hired a small room in Boston for the purpose of selling old and new books at auction, in the long winter nights. He understood his business, the wants and tastes of the people, and was successful; his little room began to be crowded, while the heat of the stove, the smoke of cigars, the fumes of tobacco saliva, and the emanations from a motley mob of human beings made the apartment a poisonous hole. The audience was different every night, and was continually changing, by individuals coming in and taking the places of those going out, but the auctioneer remained the whole time, not only breathing a vitiated atmosphere, but overstraining and overheating the voice-organs.

In this heated and exhausted condition he would go out into the cold air to his home; his mouth being open in walking, his throat and neck unprotected against the winter air; the voice-organs became diseased; presently they began to lose their power of throwing off the effects of the little colds taken from time to time, which invariably settled in the throat; meanwhile slighter causes gave colds, and longer time was required to get rid of them, and all at once he noticed that he was frequently hacking or hemming or clearing his throat; then a little tickling in the throat; next ever so slight a cough on rising in the morning. But while all this was going on, he was making a fortune, and he retired from business with a hundred thousand dollars and a consumptive cough, of which he died in less than a year after he gave up business.

This narration applies to clergymen, some of whom die every year, long before their time, from simply exposing themselves to the out-door air too soon after preaching. Some get on a horse or into a vehicle to fulfill an appointment miles away, and in the quiet condition of the body, while thus riding, and there being more or less wind in motion, the tender voice-organs are chilled in a few seconds, inducing, sometimes within an hour, more or less of clearing the throat by hemming.

In some constitutions, naturally feeble from age, or a too sedentary life, pneumonia is induced by such an exposure, followed by death in a few days. One of the most eminent and able clergymen of this generation thus perished not long ago. If men do not learn and obey the laws of our being, a miracle will not be wrought to save them from the penalty. Even in summer time it is dangerous to ride immediately after delivering an address; take an hour to cool off slowly, and eat something first, for instance, a good warm dinner.

